# Writing a Successful Grant: Tips and Tools

**Published:** 2015-05-01

**Authors:** Constance Visovsky

**Affiliations:** University of South Florida, College of Nursing, Tampa, Florida

As an oncology advanced practitioner, you may find yourself called upon to write or partner with a nurse scientist to write a grant to support or improve educational, patient care, or professional activities. There are different types of grants that can be used to support a variety of activities, such as research grants, educational program development grants, and quality improvement/clinical practice grants, to name a few.

## GRANT PREPARATION

Writing your grant application requires quite a bit of preparation. Before you begin, it is important to review the grant application and its specific instructions—they can vary widely in their parameters. Adherence to the grant instructions is a critical aspect of grant preparation. Grant proposals that do not adhere to page length, font type, and other specifications may not even be reviewed.

Pay careful attention to the grant personnel who will carry out the proposal. Some grant mechanisms require a doctoral degree as the minimum preparation for the primary investigator or for educational grants, the program director. You must consider what additional expertise may be required to carry out the project or research successfully. You may need a nurse clinician or other provider to grant access to a patient population, a statistician, or another specialty (physical therapy, laboratory services, educator) that would provide expertise and services to complete the project or research.

Data analysis for the research or project outcomes should be considered at the very start of the proposal. Secure the advice of a statistician in determining the study sample size, measures to be used, and the plan for data analysis.

As protection of human subjects and informed consent are of paramount importance, even in simple survey or educational research methods, a thorough understanding of Internal Review Board (IRB) requirements and submission for IRB approval requires planning; it can take several months to gain approval. Lastly, consideration of budget costs and constraints is necessary to be sure to be able to cover the costs of completing the proposed project or research.

## GRANT WRITING

Clarity and attention to detail are aspects of grant writing that make for a successful application. Be clear, concise, and precise. Define what you are proposing, for whom it is intended, and exactly how it will be accomplished. For those new to grant writing, seek out an experienced mentor to assist you in the development of your ideas and the review of the drafts of the proposal prior to submission. The National Institutes of Health peer review criteria for scoring grants is helpful when considering the areas a well-written grant addresses.

Your proposal should speak to the significance of the issue or problem to be addressed and how, if the aims are achieved, the findings may change the field to improve clinical practice or the care of patients. The review of the investigative team should explicate the appropriateness of the team members for the project or research and show that they have a track record of accomplishments that make them ideal partners for your work. Innovation in clinical practice and approaches or methods that are novel or result in a paradigm shift are quite appealing. The approach should be well specified, detailed, and well reasoned, with consideration of burden to the subject or population. Lastly, be sure to demonstrate that the environment for the conduct of the project or research is supportive and that resources are available to support the successful completion of the grant.

## RESOURCES

There are many reliable online resources available for both grant-writing support and available funding. You will find a good number of such resources listed in the Table to the left. But don’t neglect your own institution for funding information as well. Many institutions have foundations for grant support of approved projects.

**Table 1:**
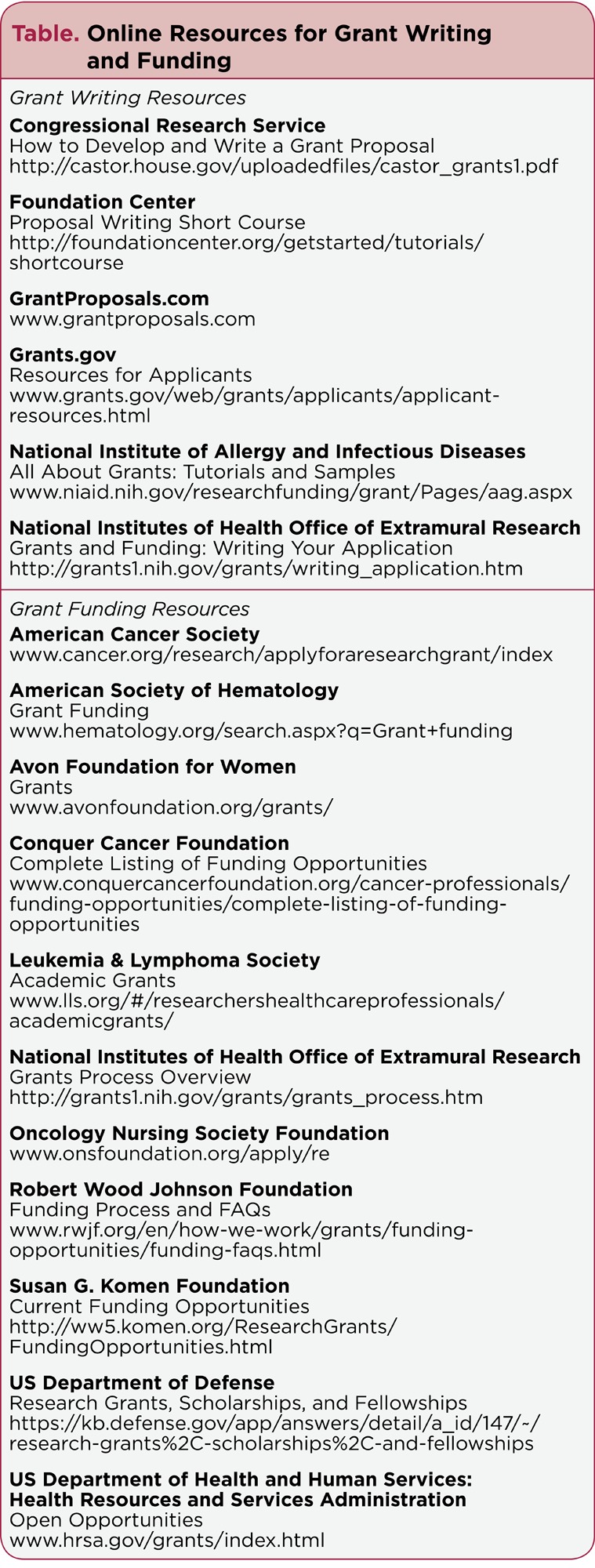
Online Resources for Grant Writing and Funding

## CONCLUSION

Grant writing can be a daunting process. But if you approach it step by step and stay positive and focused, it will not be nearly as difficult as you might imagine. Remember, the more preparation and attention to detail you put in, the more likely that you will have success in securing your desired funding.

